# Tick-borne encephalitis in adults in Denmark: a nationwide prospective cohort study from 2015 to 2023

**DOI:** 10.1007/s00415-025-12986-5

**Published:** 2025-03-03

**Authors:** Anna Maria Florescu, Thomas Bryrup, Carsten Schade Larsen, Lykke Larsen, Lothar Wiese, Hans Rudolf Lüttichau, Micha Phill Grønholm Jepsen, Birgitte Rønde Hansen, Christian Østergaard, Anja Vad Søndergaard, Peter H. S. Andersen, Lasse Skafte Vestergaard, Ria Lassaunière, Anders Fomsgaard, Bo Bødker Jensen, Jacob Bodilsen, Henrik Nielsen, Anne-Mette Lebech, Helene Mens

**Affiliations:** 1 Department of Infectious Diseases, Copenhagen University Hospital – Rigshospitalet, Esther Møllers Vej 6, 2100 Copenhagen, Denmark; 2 Department of Infectious Diseases, Aarhus University Hospital, Aarhus, Denmark; 3 Department of Infectious Diseases, Odense University Hospital, Odense, Denmark; 4 Department of Infectious Diseases, Zealand University Hospital, Roskilde, Denmark; 5 Department of Infectious Diseases, Copenhagen University Hospital – Herlev and Gentofte, Herlev, Denmark; 6 Department of Pulmonology and Infectious Diseases, Copenhagen University Hospital – North Zealand, Hillerød, Denmark; 7 Department of Infectious Diseases, Copenhagen University Hospital – Amager and Hvidovre, Hvidovre, Denmark; 8 Department of Clinical Microbiology, Copenhagen University Hospital – Amager and Hvidovre, Hvidovre, Denmark; 9 Diagnostic Infectious Disease Preparedness, Statens Serum Institut, Copenhagen, Denmark; 10 Department of Infection Epidemiology and Prevention, Statens Serum Institut, Copenhagen, Denmark; 11 Department of Virus and Microbiological Special Diagnostics, Statens Serum Institut, Copenhagen, Denmark; 12 Department of Clinical Microbiology, Zealand University Hospital – Køge, Slagelse, Denmark; 13 Department of Infectious Diseases, Aalborg University Hospital, Aalborg, Denmark; 14 Department of Clinical Medicine, Aalborg University, Aalborg, Denmark; 15 Institute of Clinical Medicine, University of Copenhagen, Copenhagen, Denmark

**Keywords:** Tick-borne encephalitis, TBE, Clinical characteristics, Outcome, Test activity, Herpes simplex encephalitis

## Abstract

**Background:**

Our aim was to characterize the clinical presentation and outcome in adults with tick-borne encephalitis (TBE) and to determine the incidence and test activity of TBE in Denmark.

**Methods:**

A nationwide prospective cohort study of all adults hospitalized with TBE at departments of infectious diseases in Denmark from 2015 to 2023. An age- and sex-matched cohort of herpes simplex virus type 1 (HSV-1) encephalitis patients was included to compare outcome.

**Results:**

Fifty-two patients with TBE were included. Median age was 50 years, 32/52 (62%) were men, 1/52 (2%) was fully vaccinated against TBE at the time of infection, 29/52 (56%) were infected in Denmark. Upon admission 25/52 (48%) had meningitis, 27/52 (52%) encephalitis, three of the latter 3/52 (6%) with additional myelitis or radiculitis. Admission to the intensive care unit 6/52 (12%) and death 2/52 (4%) were associated with pre-existing comorbidities and older age. At 3-month follow-up, 16/50 (32%) had an unfavorable outcome (Glasgow Outcome Scale score 1–4) compared to 39/52 (75%) in the HSV-1 cohort. The most common residual symptoms at 6-month follow-up or later were headache, cognitive impairment, and fatigue. The TBE incidence increased from 0.03/100,000 in 2015 to 0.48/100,000 in 2023, and the test rate from 5.5/100,000 in 2015 to 14.4/100,000 in 2023, with a positivity rate of 0.6% in 2015 and 3.3% in 2023.

**Conclusion:**

The incidence of TBE in Denmark increased in the study period, with clinical characteristics and outcome of adult patients comparable to reports from other European countries.

**Supplementary Information:**

The online version contains supplementary material available at 10.1007/s00415-025-12986-5.

## Introduction

Tick-borne encephalitis (TBE) is an infection of the central nervous system (CNS) caused by the tick-borne encephalitis virus (TBEV)—an infection of increasing public concern [1].

TBEV is mainly transmitted to humans by infected ticks [2]. Three clinically important TBEV subtypes exist: the European (TBEV-Eu), the Siberian (TBEV-Si), and the Far Eastern (TBEV-FE) [3]. TBEV-Eu, transmitted by ticks of the species *Ixodes Ricinus*, seems to have a milder clinical course compared to the other subtypes and is asymptomatic in approximately two-thirds of the cases [2, 4]. A Danish study showed that 20% of forestry workers on the island of Bornholm, an endemic TBEV area, were seropositive without recollection of symptoms [5]. Symptomatic cases usually have a biphasic course with an initial flu-like phase. After a shorter symptom-free period, typically of a week, around one-third develop a second phase infection of the CNS [2]. This infection ranges from mild meningitis to severe encephalitis, with or without myelitis or radiculitis [6]. In adults, the case-fatality rate of TBEV-Eu is reported to be about 1% and up to 50% experience long-lasting neurological sequelae [7]. TBE can be prevented by avoiding ticks and by vaccination. As no specific antiviral treatment exists, hospitalized patients are treated symptomatically [8].

In recent years, the incidence of TBEV infections has increased in Europe, likely due to climate change prolonging the transmission season and expanding endemic areas, as well as an increase in outdoor activities, especially during the COVID-19 pandemic [9–11]. A total of 3650 TBEV cases were reported from European Union/European Economic Area (EU/EEA) countries in 2022 [12]. The majority were diagnosed from June to November, when the ticks are most active [2, 12].

In Denmark, TBE has been observed since the 1950s, limited to a few (one to five) yearly cases on Bornholm [4]. In 2008–2009, the first cases outside Bornholm were identified, and in 2019, a new risk area was established in North Zealand. In November 2023, TBE became a notifiable disease, with cases increasing to 28 that year [13]. TBE diagnostics and national reporting to the European Centre for Disease Prevention and Control’s (ECDC) surveillance system (TESSy) are centralized at Statens Serum Institut (SSI). SSI is responsible for monitoring epidemic diseases and providing risk estimations. The increase in TBE cases and new geographical risk areas led to public concern and an unprecedented vaccine demand, and in June 2023, SSI reported having delivered a year’s normal consumption of vaccines within a few weeks [14].

The aim of this study was to make a first-time description of the disease course in adult patients hospitalized with TBE in Denmark and determine the yearly incidence and test activity of TBE from 2015 to 2023. This description includes clinical characteristics, objective findings, diagnostic workup, and outcome in terms of sequelae. Outcome was compared to a matched cohort of patients hospitalized with herpes simplex virus type 1 (HSV-1) encephalitis to contextualize the course of TBE.

## Materials and methods

### Study design

We performed a nationwide prospective cohort study of all adults hospitalized with TBE at departments of infectious diseases in Denmark between 1st of January 2015 and 31st of December 2023.

### Setting

In December 2023, the total population of Denmark numbered 5.9 million people. In Denmark, healthcare is tax-financed and provided to all residents free of charge. A unique ten-digit personal identification number is assigned to all Danish residents at birth or upon immigration and can be used for linkage of all healthcare information at an individual level.

### Data sources

The Danish Study Group of Infections of the Brain (DASGIB) is a nationwide, population-based, prospective cohort study enrolling all patients ≥ 18 years with a CNS infection managed by departments of infectious diseases in Denmark since 2015 [15]. We used the DASGIB database to identify all adults hospitalized with TBE in the inclusion period. Additionally, a cohort of matched controls with HSV-1 encephalitis was identified. Subjects were matched 1:1 on sex and age ± 5 years. One HSV-1 patient was used as a control for two different TBE patients due to lacking matches.

TBE diagnostics (polymerase chain reaction (PCR) and serology) are centralized at SSI. TBEV-IgM and –IgG antibodies in serum and/or cerebrospinal fluid (CSF) were measured using the Serion FSME/TBE Virus ELISA (Serion Diagnostics, Germany) following instructions of the manufacturer. TBEV-RNA in serum and/or CSF was detected with real-time PCR.

To calculate the yearly incidence of TBE from 2015 to 2023, numbers on all second phase TBE cases, including sex and age (including patients < 18 years), were retrieved from SSI [13]. Numbers on the yearly Danish population including sex and age were retrieved from Statistics Denmark, the central authority on Danish statistics [16].

To calculate the yearly test rate of TBE from 2015 to 2023, data on TBE tests were retrieved from The Danish Microbiology Database (MiBa) (including patients < 18 years). MiBa is a nationwide, automatically updated database of all microbiological test results in Denmark [17]. Every tested person only appeared once, regardless of the number of tests, per calendar year from 2015 to 2023.

### Study population

Study participants fulfilled the DASGIB criteria for CNS infections [15]. Meningitis was defined as a clinical presentation consistent with viral meningitis (e.g., headache, neck stiffness, photophobia, hyperacusis, fever) without signs of encephalitis. Encephalitis was defined according to the International Encephalitis consortium [18]. Meningoencephalomyelitis/-radiculitis was defined as encephalitis with involvement of the spinal cord or spinal nerve roots. Pleocytosis was defined as ≥ 10 × 10^6^ cells/L in the CSF [15].

Patients with TBE were defined by either a) detection of TBEV-IgM and -IgG antibodies in serum, b) TBEV-IgM in CSF, c) seroconversion or four-fold increase of TBEV-specific antibodies in paired serum samples, or d) detection of TBEV RNA by PCR in blood or CSF [19, 20].

Patients with HSV-1 encephalitis had a clinical presentation consistent with encephalitis and at least one of the following: a) detection of HSV-1 DNA in CSF by PCR or b) positive HSV-1 intrathecal antibody index test [21].

### Variables

#### TBE patients

We obtained data on date and place of admission, basic demographics (age, sex, physical and functional status before admission), travel history, vaccination status for TBE and other flaviviruses (dengue, yellow-fever, and Japanese encephalitis virus), Charlson Comorbidity Index (CCI) score [22], immunodeficiency, history of tick bite, geographic location for tick bite, duration of symptoms, defined time from onset of symptoms to admission to hospital, clinical presentation, length of hospital stay, admission to the intensive care unit (ICU), and diagnostic work-up (biochemical and microbiological analyses of blood and CSF, and neuroimaging). Data on vaccination status were retrieved from the Danish Vaccination Register [23]. Immunodeficiency was defined as receiving treatment with immune-suppressive chemotherapy or corticosteroids, solid or hematological cancer, alcohol abuse, diabetes mellitus, congenital or acquired immunodeficiency, including human immunodeficiency virus infection [15].

The clinical presentation of TBE was classified as mild, moderate, or severe in accordance with previously published classifications [24]. Mild disease was defined as symptoms consistent with viral meningitis. Moderate disease was defined as slightly altered consciousness and/or diffuse or focal neurological symptoms. Severe disease was defined as altered consciousness and/or multifocal neurological symptoms. All patients with signs of encephalitis were classified as having moderate or severe disease.

Outcome was measured as the Glasgow Outcome Scale (GOS) score: (1) Death, (2) A vegetative state, (3) Severe sequelae and dependency upon others in daily life, (4) Moderate sequelae but with the ability to live independently, and (5) No or only mild sequelae [25]. GOS score 1–4 was categorized as an unfavorable outcome. GOS score was registered at three timepoints: Discharge, 1-month, and 3-month follow-up. In case of a missing value, the GOS score at the previous timepoint was carried forward, if the patient had a favorable outcome (i.e., GOS score 5) or death (i.e., GOS score 1). Furthermore, information on self-reported residual symptoms in terms of fatigue, headache, cognitive impairment (memory and concentration), irritability, impaired hearing or hyperacusis, and objective residual symptoms such as speech disturbances, paresis, and paresthesia were retrieved at 6-month follow-up or later.

#### HSV-1 patients

We obtained data on date and place of admission, basic demographics, CCI score, immunodeficiency, admission to the ICU, diagnostic work-up in terms of neuroimaging, and GOS score at discharge, 1-month, and 3-month follow-up.

### Statistical analysis

Categorical variables were reported as proportions and percentages, and continuous variables as medians, with interquartile ranges (IQR). The difference in age means was analyzed with an unpaired t-test. Differences in the likelihood of an unfavorable outcome (GOS score 1–4) between the TBE cohort and the HSV-1 cohort were evaluated at three timepoints (discharge, 1-month, and 3-month follow-up) using a logistic regression model accounting for age and sex. Analyses were repeated exclusively on the subgroup of TBE patients with encephalitis and their respective matched HSV-1 case. All analyses were performed using R (version 4.3.2) [26]. Logistic regressions were done with the *glm* function from the *stats* package [27]. Incidence rates, test rates, and positivity rates were calculated based on SSI records of all TBE cases in the study period. The incidence rates were adjusted for the age and sex distribution in the population.

### Ethical considerations

The DASGIB database is approved by the Danish Board of Health (record numbers 3–3013-2579/1), and this study is approved by The Danish Data Protection Agency (record number R-23066339). Patient consent or an approval from the local Ethics Committee is not needed for this type of study in Denmark. All data are managed in compliance with relevant data protection and privacy regulations and in accordance with the Helsinki declaration.

## Results

A total of 52 adult patients with TBE were identified in the DASGIB cohort (Table 1). Consistent with tick activity, peak months of diagnosis were June through August with 36/52 (69%) cases. In 29/52 (56%) cases, the infection was suspected to be acquired in Denmark, with Bornholm (9/29; 31%) and North Zealand (15/29; 52%) as the most common locations. Remaining cases were mainly imported from Sweden (15/23; 65%). The distribution of number of cases infected in Denmark versus imported cases was constant throughout the study period.

**Table 1:** Baseline characteristics of 52 adult patients with tick-borne encephalitis, diagnosed between 2015 and 2023, in Denmark

Characteristics	TBE cohort (*N* = 52)
	No. (%) of patients or median (IQR)
Age, years	50 (38–59)
Sex, male	32 (62)
Admission year	
2015–2018	8 (15)
2019–2023	44 (85)
**Region of Denmark (place of diagnosis)**	
Zealand	40 (77)
Funen	6 (12)
Jutland	6 (12)
**Exposure and vaccination**	
History of tick bite	36 (69)
Suspected infection site	
Denmark	29 (56)
Abroad: Sweden (*N* = 15), Baltic (*N* = 4), Poland (*N* = 4)	23 (44)
Prior TBE-vaccination	2 (4)
Fulfilling criteria for TBE-vaccination^a^	14 (27)
**Disease course**	
Biphasic course of TBE	37 (71)
Days from tick bite to first phase	3 (2–6)
Days between onset of first phase to onset of second phase	14 (10–16)
Days from admission to testing for TBE (*N* = 51)	1 (0–2)
**First phase symptoms**	
Fever	30 (81)
Headache	18 (49)
Myalgia and/or arthralgia	12 (32)
Abdominal symptoms (diarrhea, nausea, vomiting)	6 (16)
**Second phase**	
Encephalitis	27 (52)
Meningoencephalomyelitis/-radiculitis Moderate disease Severe disease	3 (6) 7 (13) 20 (38)
Meningitis	25 (48)
**Second phase symptoms**	
Fever	47 (90)
Headache	46 (88)
Nausea	29 (56)
Confusion	18 (35)
Gait disturbance	15 (29)
Focal neurological deficits	10 (19)
Aphasia or speech latency	9 (17)
Ataxia	4 (8)
Personality changes	3 (6)
Seizures	0

*IQR* interquartile range; *TBE* tick-borne encephalitis. Categorical variables are presented as n/N (%), and continuous variables as medians with interquartile rates (IQRs)

^a^ Due to frequent, prolonged stays in risk areas, in accordance with the national recommendations for TBE vaccination (https://www.ssi.dk/vaccinationer/vaccineleksikon/c/fsme-vaccine)

### Baseline characteristics

The median age was 50 years (IQR 38–59), and 32/52 (62%) were men (Table 1). A total of 49/52 (94%) had no physical/cognitive deficits prior to admission, and 41/52 (79%) were working/studying full-time. Median CCI score was 1 (IQR 0–2), and 15/52 (29%) had a CCI score ≥ 2. One patient was immunosuppressed due to chronic lymphocytic leukemia (CLL). A total of 37/52 (69%) reported a tick bite prior to symptom debut. Only 2/52 (4%) were previously TBE vaccinated. One had received a single dose a month prior to the infection. The other, a 76-year-old woman, had three doses with the last dose a year before the infection and was regarded as a vaccine failure. Median duration of hospitalization was 7 days (IQR 5–11 days). Median time from onset of first phase to TBE testing was 18 days (IQR 16–22 days), and from admission to TBE testing was 1 day (IQR 0–2 days).

### Clinical features of TBE

Based on the clinical presentation and disease course, 25/52 (48%) patients were diagnosed with meningitis, and 27/52 (52%) with encephalitis (Table 2). The groups had a median age of 42 and 55 years (p = 0.002), respectively. Three of the patients with encephalitis 3/52 (6%) had additional myelitis or radiculitis (thus, meningoencephalomyelitis/-radiculitis). Of the 27 patients with encephalitis, 7/52 (13%) were classified as having severe disease. A biphasic disease course was reported in 37/52 (71%). Median time from tick bite to primary phase was 3 days (IQR 2–6), and between onset of first to second phase 14 days (IQR range 10–16). Most common primary phase symptoms were fever 30/37 (81%), headache 18/37 (49%), and myalgia/arthralgia 12/37 (32%), and in second phase fever 47/52 (90%) and headache 46/52 (88%). Notably, no patients presented with seizures.

**Table 2:** Baseline characteristics with pre-admission morbidity and outcome for 52 adult patients with tick-borne encephalitis, and 52 matched adult patients with herpes simplex virus type 1 encephalitis, diagnosed between 2015 and 2023, in Denmark

Characteristics	TBE cohort (*N* = 52)	HSV-1 cohort (*N* = 52)
	No. (%) of patients or median (IQR)	
Age, years	50 (38–59)	51 (38–61)
Sex, male	32 (62)	32 (62)
**Type of CNS-infection**		
Encephalitis	27 (52)	52 (100)
Viral meningitis	25 (48)	0
**Comorbidity**		
Immunosuppression ^a^	1 (2)	11 (21)
CCI 0–1	37 (71)	30 (58)
CCI 2 or more	15 (29)	22 (42)
**Physical status before infection**		
No physical/cognitive deficits	49 (94)	40 (77)
Mild physical/cognitive deficits	2 (4)	9 (17)
Moderate physical/cognitive deficits	1 (2)	2 (4)
Severe physical/cognitive deficits	0	1 (2)
**Functional status before infection**		
Full-time work/study	41 (79)	31 (60)
Part-time work/study	0	6 (12)
Unemployed	1 (2)	2 (4)
Disability pension/sick leave	1 (2)	1 (2)
Retired, totally independent	8 (15)	9 (17)
Retired, dependent on some help	1 (2)	3 (6)
ICU admission	6 (12)	20 (38)
MRI brain with lesions attributed to infection	8 (22)	44 (85)
Death related to infection	2 (4)	5 (10)

*CNS* central nervous system; *CCI* Charlson Comorbidity Index; *IQR* interquartile range; *TBE* tick-borne encephalitis; *HSV-1* herpes simplex virus type 1; *ICU* intensive care unit; *MRI* magnetic resonance imaging. Categorical variables are presented as n/N (%), and continuous variables as medians with interquartile rates (IQRs)

^a^ Alcohol abuse, diabetes mellitus, solid or hematological cancer (including malignant melanoma, excluding other skin cancers), congenital or acquired immunodeficiency, including human immunodeficiency virus infection

^b^ The TBE cohort was missing data for one patient at 1-month follow-up (thus *N* = 51) and for two patients at 3-month follow-up (thus *N* = 50)

### Paraclinical findings—imaging results

Magnetic resonance imaging (MRI) of the brain ± spinal cord was performed in 37/52 (71%) (Table 2). In 8/37 (22%), MRI showed lesions attributed to TBE such as leptomeningeal enhancement and meningoencephalomyelitis/-radiculitis. Remaining scans were without new pathological changes.

### Paraclinical findings—CSF and peripheral blood

Lumbar puncture was performed in 51/52 (98%) patients (Table 3). The patient without lumbar puncture had symptoms compatible with meningitis and was diagnosed due to rising TBEV antibody titers in serum. The median CSF leucocyte count was 74 × 10^6^ cells/L (IQR 49–123 × 10^6^ cells/L) with a mononuclear median cell count of 52 × 10^6^ cells/L (IQR 32–94 × 10^6^ cells/L), and a median CSF protein of 0.68 g/L (IQR 0.54–0.83 g/L). Four patients did not have CSF pleocytosis with lumbar puncture performed 7, 19, 22, and 30 days after onset of the first phase, respectively. Two of these patients developed meningitis, and two encephalitis.

**Table 3:** Paraclinical data among 52 adult patients with tick-borne encephalitis, diagnosed between 2015 and 2023, in Denmark

Laboratory results	No. (%) of patients or median (IQR)
**Cerebrospinal fluid**	
CSF, leucocytes (10^6^/L) (*N* = 51) ^a^	74 (49–123)
CSF, mononuclear leucocytes (10^6^/L) (*N* = 51)	52 (32–94)
CSF lactate (mmol/L) (*N* = 41)	2.2 (2–2.6)
CSF glucose (mmol/L) (*N* = 51)	3.5 (3.25–3.8)
Glucose index (*N* = 50)	0.55 (0.50–0.63)
CSF protein (g/L) (*N* = 48)	0.68 (0.54–0.83)
**TBE antibodies or RNA in CSF**	
TBE IgM positive only (*N* = 51)	29 (57)
TBE IgM and IgG positive (*N* = 51)	2 (4)
TBE RNA positive (*N* = 34)	2 (6)
**Serum**	
B-leucocytes (10^9^/L) (*N* = 52)	11 (9–13)
C-reactive protein (mg/L) (*N* = 52)	7 (3–18)
**TBE antibodies or RNA in serum**	
TBE IgM and IgG positive (*N* = 52)	51 (98)
TBE RNA positive (first phase suspected) (*N* = 7)	2
**Diagnostic imaging** MRI brain ± spinal cord (*N* = 37)	37 (71)
With lesions attributed to TBE infection	8 (22)
With vascular changes ^b^	5 (14)
Other ^c^	6 (16)

*CSF* cerebrospinal fluid; *IQR* interquartile range; *TBE* tick-borne encephalitis; *IgM* immunoglobulin M; *IgG* immunoglobulin G; RNA, ribonucleic acid. Categorical variables are presented as n/N (%), and continuous variables as medians with interquartile rates (IQRs)

^a^ Four patients had < 10 × 10^6^ leukocytes/L in CSF (3, 7, 7, and 9 cells, respectively)

^b^ Three patients had white matter lesions, one with 2 small artefacts in cerebellum (described as most likely cavernous hemangiomas)

^c^ Insufficient imaging; asymmetric lateral ventricles; no signs of encephalitis; previous left-sided craniotomy with stationary substance loss in the frontal left hemisphere; sinusitis; stationary sequelae following right-sided temporal lobe bleeding and substance loss; ventricular dilation; no focal changes

Blood samples at admission showed mild systemic inflammation with a median leucocyte count of 11 × 10^9^ cells/L (IQR 9–13 × 10^9^ cells/L), and a median C-Reactive Protein (CRP) of 7 mg/L (IQR 3–18 mg/L).

### Paraclinical findings—TBEV RNA and TBEV antibodies in serum and CSF

All patients underwent serologic testing with 51/52 (98%) being serum TBEV-IgM and -IgG positive (Table 3). The seronegative patient had received high-dose prednisolone, plasmapheresis, and intravenous immunoglobulin (IVIG) prior to testing due to suspected paraneoplastic Guillain Barré syndrome and was afterwards diagnosed based on the presence of TBEV-IgM in CSF. Seven patients were tested for TBEV RNA by PCR in serum, two were positive. Both patients were in the first phase at the time and were later readmitted with CNS symptoms. Analysis for CSF TBEV antibodies was performed in 36/52 (69%) with 29/36 (81%) being IgM positive/IgG negative, and 2/36 (6%) being IgM and IgG positive. A total of 34/52 (65%) were tested for TBEV RNA by PCR in CSF, 2/34 (6%) were positive. A *Borrelia burgdorferi* (*Bb*) s.l. intrathecal antibody index was measured in 44/52 (85%) of the patients at the time of lumbar puncture, none were positive.

### Concomitant diagnoses and treatment

A total of 6/52 patients (12%) were admitted to the ICU, and 5/52 (10%) received immunomodulating treatment (high-dose prednisolone, plasmapheresis, and/or IVIG). Treatment was either due to a suspected autoimmune condition (anti-N-methyl-d-aspartate receptor (anti-NMDAR) encephalitis, secondary autoimmune reaction to TBE, or paraneoplastic Guillain- Barré syndrome) or was symptomatic (Supplementary Table 1).

### Outcome

**Fig. 1:**
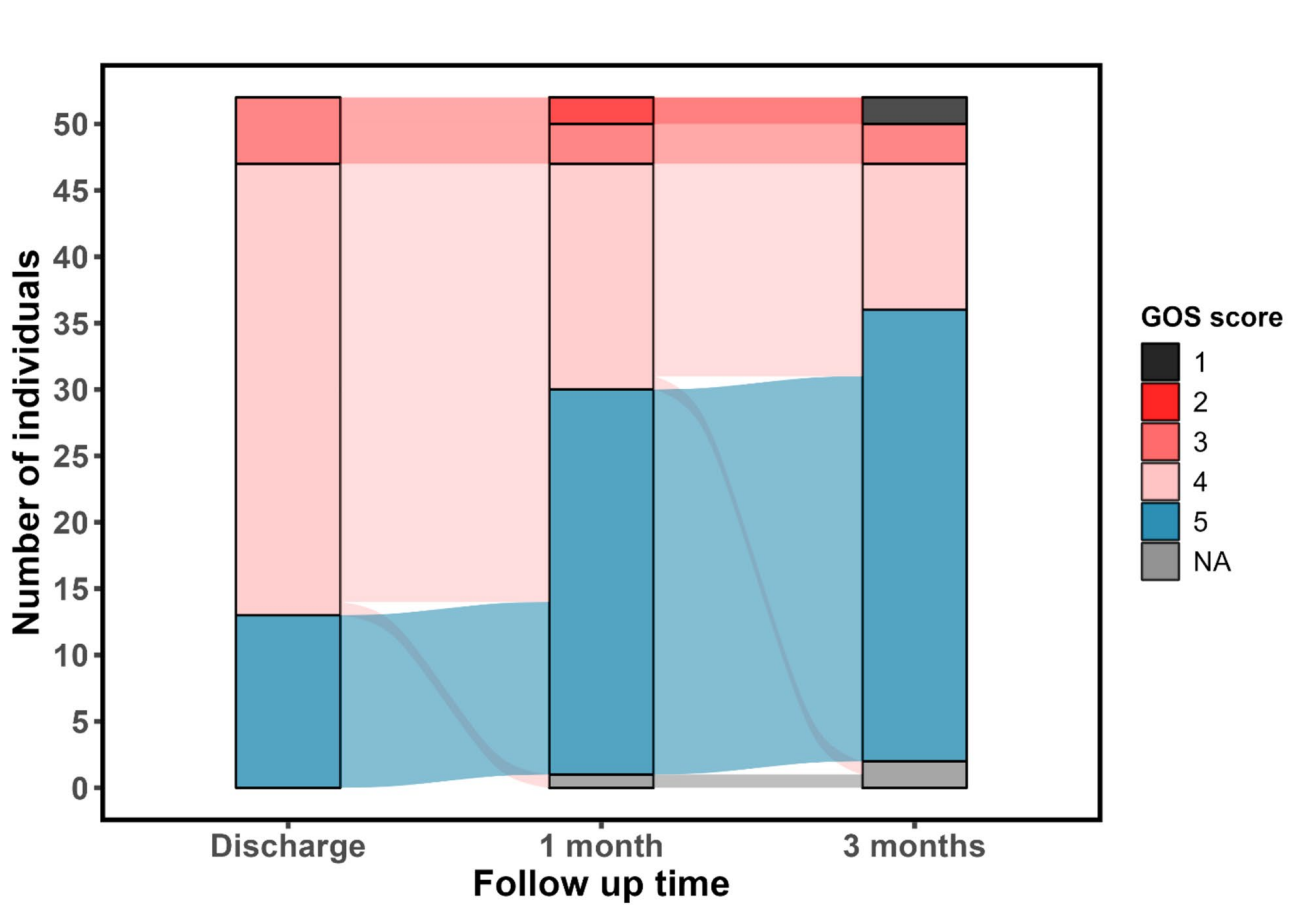
Sankey diagram showing outcome measured as Glasgow Outcome Scale (*GOS*) score over time in 52 patients diagnosed with tick-borne encephalitis in Denmark from 2015 to 2023. The GOS score is divided into: (1) Death, (2) A vegetative state, (3) Severe sequelae and dependency upon others in daily life, (4) Moderate sequelae but with the ability to live independently, and 5) No or only mild sequelae. GOS score 1–4 was categorized as an unfavorable outcome. At 1-month follow-up, one patient was missing data (thus *N* = 51), and at 3-month follow-up, two patients were missing data (thus *N* = 50). Notably, the diagram shows that patients with GOS score 4 at discharge could improve to GOS score 5 within 3 months, but those with GOS score 3 remained in that group or worsened. *NA* not available

**Fig. 2:**
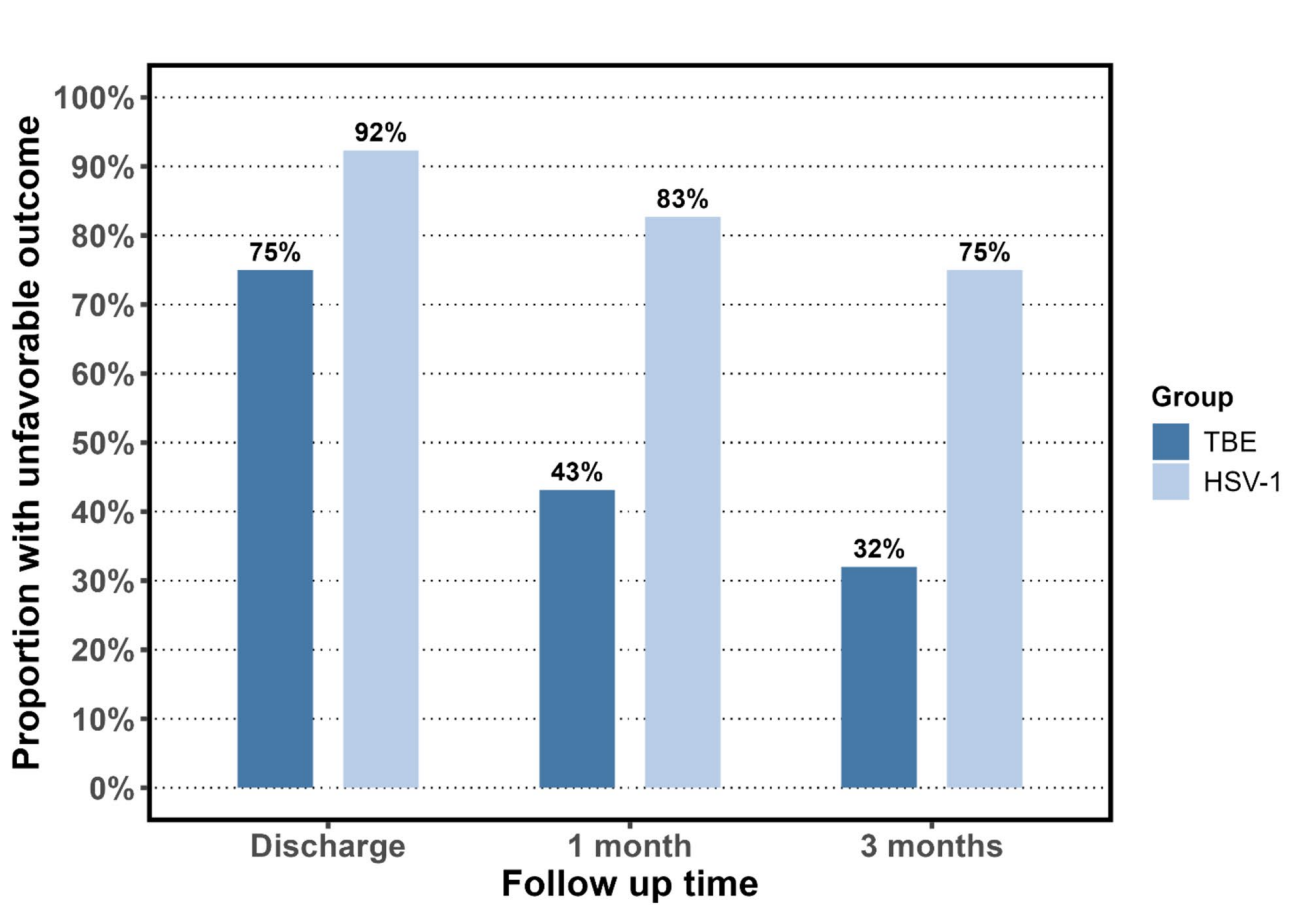
Percentage with an unfavorable outcome (Glascow Outcome Scale score 1–4) in 52 patients with tick-borne encephalitis, and in 52 patients with herpes simplex virus type 1 encephalitis, at the three timepoints: Discharge, 1-month follow-up, and 3-month follow-up. The tick-borne encephalitis cohort had missing data for one patient at 1-month follow-up (thus *N* = 51) and for two patients at 3-month follow-up (thus *N* = 50). *TBE* tick-borne encephalitis; *HSV-1* herpes simplex virus type 1

Case-fatality was 2/52 (4%). Both patients were men, aged 66 and 68 years, with CCI scores of 5 and 4, respectively. One was diagnosed with CLL. A total of 19/52 (37%) patients were discharged to neurorehabilitation (Supplementary Table 1). The GOS score was unfavorable in 39/52 (75%) at discharge, 29/51 (57%) at 1-month follow-up, and 16/50 (32%) at 3-month follow-up (Figs. 1 and 2). Encephalitis was associated with a worse outcome compared to meningitis. The GOS score at 3-month follow-up was unfavorable in 14/27 (52%) in the encephalitis group compared to 4/25 (16%) in the meningitis group. Of the 41/52 (79%) working or studying full-time before diagnosis, 24/41 (59%) returned to their occupation from a month up to 15 months after diagnosis. A total of 21/52 (40%) patients were seen at a 6-month follow-up or later and reported residual symptoms such as headache (7/21, 33%), cognitive impairment (6/21, 29%), fatigue (5/21, 24%), and impaired hearing or hyperacusis (3/21, 14%) (Supplementary Table 1).

### Comparison of outcome to patients with herpes simplex virus type 1 encephalitis

Median age in the HSV-1 encephalitis cohort was 51 years (IQR 38–61), and 32/52 (62%) were men (Table 2). A total of 40/52 (77%) had no physical/cognitive deficits prior to admission, and 5/52 (10%) of the patients died. A total of 44/52 (85%) had MRI lesions attributed to the infection (i.e., unilateral or bilateral lesion in the medial temporal lobe and inferolateral frontal lobe). The GOS score was unfavorable in 48/52 (92%) at discharge, 43/52 (83%) at 1-month, and 39/52 (75%) at 3-month follow-up (Fig. 2).

A logistic regression model accounting for sex and age showed a higher likelihood of an unfavorable GOS score (GOS 1–4) among HSV-1 patients compared to the TBE patients. At discharge, 1-month, and 3-month follow-up, the odds ratio (OR) of an unfavorable GOS score in the HSV-1 cohort compared to TBE cohort were 4.1 (95% CI 1.2–14), 6.5 (95% CI 2.6–16.2), and 6.9 (95% CI 2.8–16.9), respectively. Sex and age were not significant.

A subgroup analysis only considering TBE cases with encephalitis and their matched HSV-1 cases showed no significant difference in the OR of an unfavorable outcome in the two cohorts. At discharge, 1-month, and 3-month follow-up, the OR of an unfavorable GOS score in the HSV-1 cohort compared to TBE cohort were 2.1 (95% CI 0.2–24.4), 1.8 (95% CI 0.5–6.3), and 3.16 (95% CI 1.0–10.3), respectively.

### TBE incidence and diagnostics in Denmark from 2015 to 2023

**Fig. 3:**
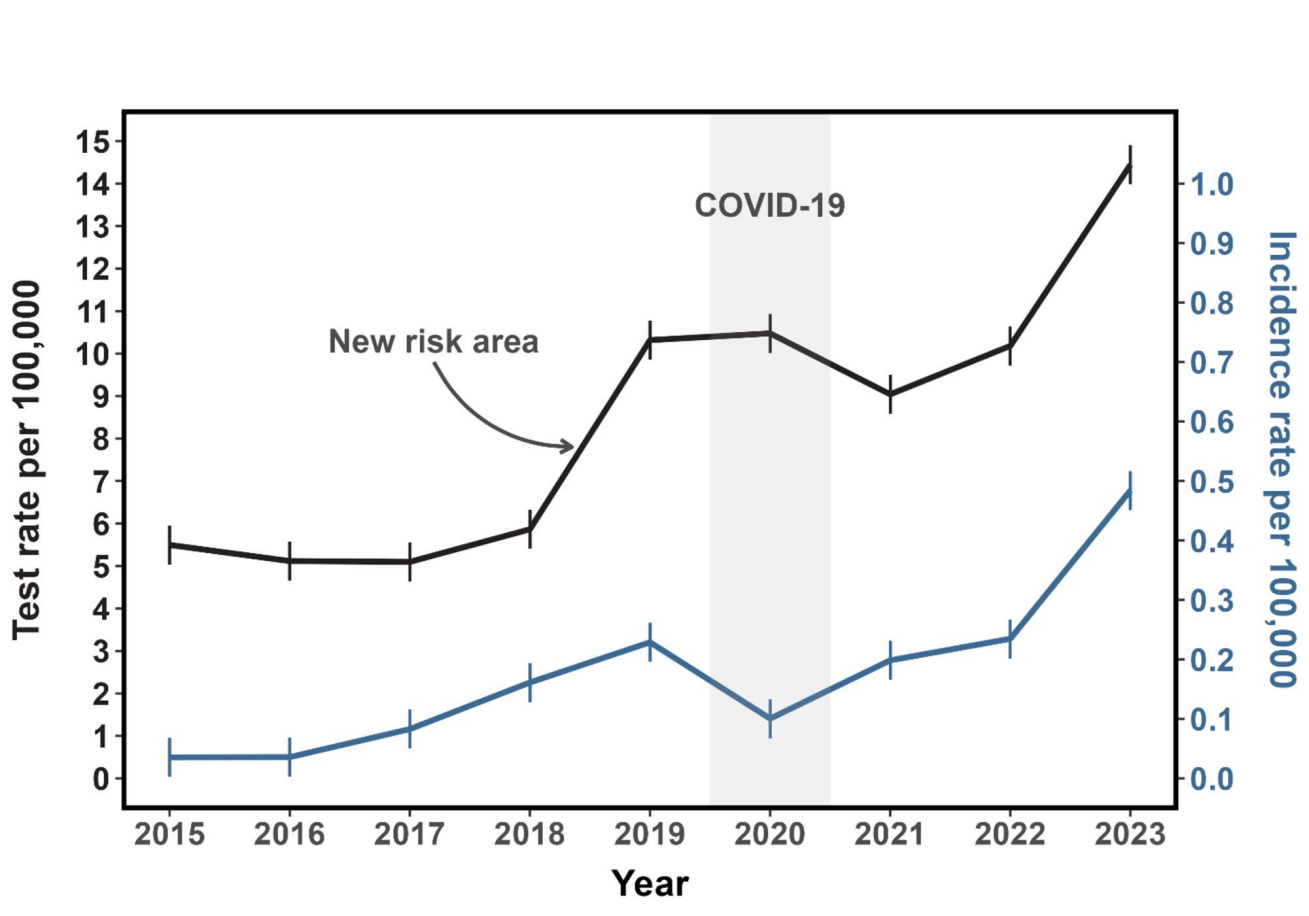
Incidence and test rates of tick-borne encephalitis in Denmark from 2015 to 2023. Data retrieved from Statens Serum Institut and Statistics Denmark. The black line represents the test rate (per 100,000 individuals), and the blue line represents the incidence rate, scaled to the same axis. The shaded region highlights the possible impact of COVID-19 travel restrictions during 2020, resulting in fewer imported cases. The arrow points to an increase in test rates in 2018–2019, possibly due to the establishment of a new risk area in North Zealand in 2019

SSI reported 89 patients (hereof 4 children < 18 years) diagnosed with TBE with CNS involvement in Denmark from 2015 to 2023. The incidence of TBE increased from 0.03/100,000 individuals in 2015 to 0.48/100,000 in 2023. The incidence rate ratio was 14 (95% CI 13.6–14.4) in 2023 with 2015 as reference (Fig. 3 and Supplementary Fig. 1).

**Fig. 4:**
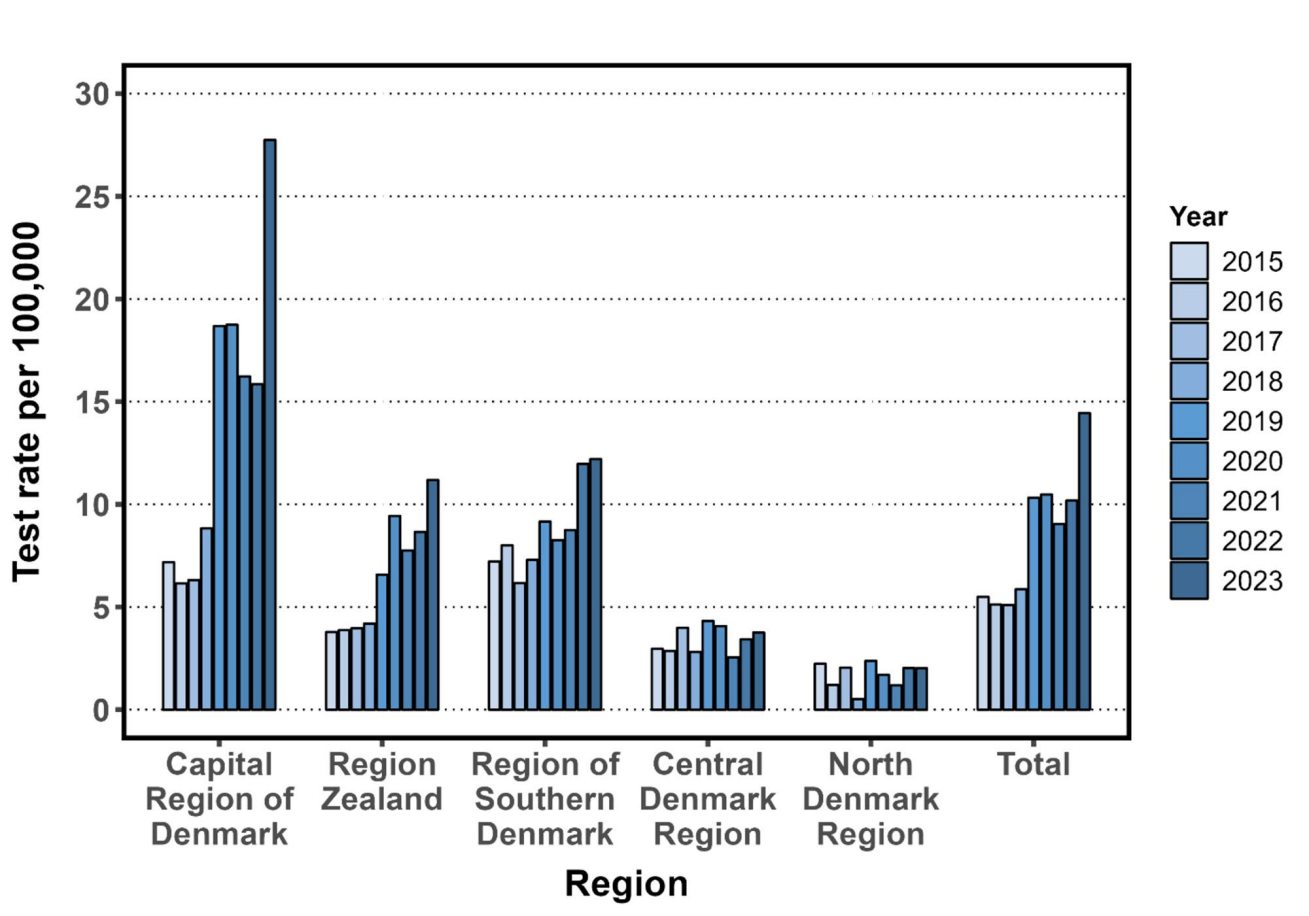
Tick-borne encephalitis test rate in the five different regions in Denmark from 2015 to 2023. Every tested person only appears once per calendar year

The test rate in the total Danish population increased from 5.5/100,000 in 2015 to 14.4/100,000 in 2023 (Fig. 3). The positivity rate increased from 0.6% in 2015 to 3.3% in 2023. The increased test activity was driven by the Capital Region of Denmark (covers Bornholm and North Zealand), and Region Zealand, as well as the Region of Southern Denmark, where locally acquired TBE cases have been diagnosed (Fig. 4). Test activity was most frequent among the 40- to the 70-year-olds, and men were tested more often than women (Supplementary Fig. 2).

## Discussion

TBE is an emerging disease of public health importance associated with significant morbidity. This is the first nationwide study in Denmark to describe the clinical characteristics and outcome of adults with TBE, as well as the TBE incidence in relation to test activity.

The clinical presentation, mortality, and outcome were in alignment with reports from other European countries. An unfavorable outcome was associated with pre-existing comorbidities and older age. TBE was associated with less mortality and morbidity compared to a matched cohort of HSV-1 encephalitis patients. Consistent with data from ECDC showing an increase in TBE incidence in EU/EEA countries after 2018, we observed an incidence rate ratio of 14 in 2023 with 2015 as ref. [12].

The clinical characteristics of this TBE cohort were similar to formerly described TBEV-Eu cohorts in terms of basic characteristics, disease presentation, morbidity, and mortality [2, 3, 6, 9, 28–30]. Approximately half of the cases in our study had encephalitis. This is a lower percentage than the results from Norway in the study by Skudal et al. (105/153, 69%), but similar to the results from Sweden in the study by Bartholdsson et al. (381/703, 54%) [9, 30]. The patients with encephalitis in our cohort were significantly older than those with meningitis. Nearly three out of four patients had a biphasic course, in alignment with previous findings, with the most common first and second phase symptoms being headache and fever [2, 6]. In contrast, only half of the TBE cases in Norway reported a biphasic course (53%) [9].

The median length of hospital stay was 7 days, which aligns with the results from Norway (7 days) and Sweden (6 days) [9, 30]. Importantly, 13% of the patients in our cohort were classified as having severe disease, in agreement with the Norwegian (14%) and Swedish results (11%) [9, 30]. In contrast, 12% of our cohort were admitted to the ICU, which was a higher percentage than in the Norwegian (5%) and Swedish (6%) studies [9, 30]. Approximately a third of the patients in our study were discharged to neurorehabilitation (highly specialized or municipal), which is less than in the German study by Nygren et al. (42%), but higher than in the Norwegian (22%) [9, 31]. Two patients had a fatal outcome, both men in their late 60 s with substantial comorbidities, in accordance with previous studies showing pre-existing comorbidities are risk factors for severe disease and death [28]. Interestingly, in one patient, the disease course of TBE was complicated by anti-NMDAR autoimmune encephalitis, a diagnosis mainly associated with HSV-1 encephalitis [32, 33]. A single similar case from Italy has previously been reported in the literature [34].

Around 20% had MRI lesions attributed to TBE (leptomeningeal enhancement and signs of meningoencephalomyelitis/-radiculitis) in alignment with previous studies [6, 9]. TBEV has a predilection for thalami, basal ganglia, cerebellum, and anterior horns of the spinal cord. However, MRI abnormalities, if present, are generally unspecific and resemble those seen in other CNS infections [35]. In contrast, HSV-1 encephalitis usually exhibits a typical pattern on MRI involving the limbic system, which our HSV-1 cohort also supported [21].

CSF showed typical moderate pleocytosis with mononuclear predominance [6]. Four patients (4/52, 8%) were without pleocytosis (with lumbar puncture 7 to 30 days after onset of first phase), and all four had a favorable GOS score at 1-month follow-up. This percentage aligns with the Norwegian cohort, where 7% of the TBE patients with CNS infection had a normal CSF white blood cell count (disease course ranging from mild to severe infection) [9]. This finding highlights the point that patients without pleocytosis should still be examined for TBEV serology if the clinical course is suggestive of TBE.

In general, patients with TBE are hospitalized when CNS symptoms manifest in the second phase of the disease. At this stage, the diagnosis relied on serology as described in other studies [6]. TBEV RNA was only detected in CSF in a few patients (6%) consistent with previous findings [36]. Thus, TBEV PCR should be reserved for immunocompromised patients, who have difficulties activating an antibody response [37]. Most cases were also tested for Lyme neuroborreliosis due to a history of tick bite. In contrast to the Norwegian study, in which a few (5%) of the TBE patients also had a positive *Bb* intrathecal antibody index, none in our cohort did, suggesting that co-infection is not common in Denmark [9]. Nonetheless, LNB is a common infection of the nervous system in Denmark with an incidence of 2.6/100,000 individuals per year [38].

The incidence of TBE increased in our study period, except for a decrease in 2020, which was likely due to COVID-19 travel restrictions resulting in fewer imported cases (Fig. 3). We also observed an increase in test rates, although less than the corresponding incidence rates, along with the positivity rates. The test rate increase in 2018–2019, without an accompanying equivalent incidence rate increase, could be due to the establishment of a new risk area in North Zealand in 2019 (Fig. 3) [4, 13]. Altogether, our data suggested that the increase in TBE cases in the study period was real rather than due to increased test activity.

In Denmark, TBE vaccination is self-financed. Currently, national guidelines recommend TBE vaccination for people walking on trails in risk areas combined with regular tick bites, or who walk outside trails in forests and scrubs during the transmission season. In this cohort, only one patient was fully vaccinated and one patient partly, whereas an additional 14 individuals were eligible for vaccination according to guidelines (i.e., living or going on vacation, hunting, doing sports (geocaching, running) or working (woodcutter) in an endemic area) [39]. Our data suggest self-perceived risk of TBE infection may be underestimated by people at risk. Increased public awareness due to media attention on TBE in Denmark has led to a raised vaccine demand in the last few years.

## Strengths and limitations

The nationwide design with complete long-term follow-up is an important strength of our study. The comparison to matched controls with HSV-1 encephalitis allowed us to contextualize the morbidity and mortality of TBE, while the matched design reduces the confounding effect of age and sex. Notably, a limitation of this comparison is that all the patients in the HSV-1 cohort had encephalitis, compared to 27/52 (52%) in the TBE cohort. Previous studies have shown that patients with encephalitis are more likely to have an unfavorable outcome than those with viral meningitis [40].

An unequal comparison between a group of encephalitis and a mixed group of meningitis and encephalitis could bias the results towards an apparent higher morbidity of HSV-1. Therefore, we performed a subgroup analysis, considering only TBE cases with encephalitis and their matched HSV-1 cases. Here, the difference in risk of an unfavorable outcome between groups disappeared. Due to the small sample size, it is important to note that these results may be underpowered, with a risk of type II error. Although the cases were matched, the HSV-1 encephalitis patients had more cognitive/physical deficits, comorbidities, and a larger proportion were immunosuppressed prior to admission, which may also impact morbidity.

A limitation of our study is that we were only able to include 52 of the 85 adult TBE cases reported by SSI during the study period. The missing 33 cases could consist of milder cases, managed in outpatient settings, or severe cases, admitted to other departments (e.g., departments of neurology), and were thus not included in the DASGIB database. Our cohort could underrepresent the mildest cases. To address this limitation, we propose a future prospective study enrolling all patients diagnosed with TBE by SSI. Another limitation is the missing GOS score in one patient at 1-month follow-up, and two patients at 3-month follow-up, which might decrease statistical power slightly. The variability in follow-up times, as well as missing follow-up times, made it difficult to summarize long-term residual symptoms for the whole cohort. Finally, it would be relevant in a future study to explore markers of cytokine/protein profiles to shed light on the drivers of CNS inflammation in TBE.

## Conclusion

In conclusion, the disease course of TBE in Denmark was comparable to the course in other European countries. HSV-1 encephalitis patients were more likely to have an unfavorable outcome than TBE patients, although this difference was not seen when we only considered TBE cases with encephalitis. From 2015 to 2023, the incidence rate of TBE increased more than the test rate. This indicated a real increase in TBE cases, and was supported by an increasing positivity rate.

## Supplementary Information

Below is the link to the electronic supplementary material.

Supplementary file1 (DOCX 193 KB)

## Data Availability

The data is not freely available, but can be provided upon request.
