# Supplementary material for: Tick-borne encephalitis in adults in Denmark: a nationwide prospective cohort study from 2015 to 2023
Source: J Neurol. 2025 Mar 3;272(3):241. doi: 10.1007/s00415-025-12986-5 (PMC11876282; doi:10.1007/s00415-025-12986-5)

**Supplementary materials**

**Supplementary Table 1.** Detailed outcome among 52 adult patients with tick-borne encephalitis, diagnosed between 2015 and 2023, in Denmark

| **Parameters** | **No. of patients with registered result** | **No. (%) of patients or median (IQR)** | |
| --- | --- | --- | --- |
| Treatment for TBE (experimental) or concurrent infections | 52 | 6 (12) | |
| Death related to TBE | 52 | 2 (4) | |
| Neurorehabilitation related to TBE | 52 | 19 (37) | |
| Municipal neurorehabilitation | 52 | 12 (23) | |
| Highly specialized neurorehabilitation | 52 | 7 (14) | |
| Status following TBE |  |  | |
| Back to previous full-time work/studies | 41 | 24 (59) | |
| Part-time work/studies | 41 | 10 (24) | |
| Unknown | 41 | 7 (17) | |
| Residual symptoms at 6-month follow-up or later |  |  | |
| Headache | 21 | 7 (33) | |
| Cognitive impairment (memory and concentration) | 21 | 6 (29) | |
| Fatigue | 21 | 5 (24) | |
| Impaired hearing or hyperacusis | 21 | 3 (14) | |
| Paresthesia | 21 | 1 (5) | |
| Paresis | 21 | 1 (5) | |
| Abbreviations: IQR, interquartile range; TBE, tick-borne encephalitis. Categorical variables are presented as n/N (%), and continuous variables as medians with interquartile rates (IQRs). | | |  |

**Supplementary Figure 1.** Incidence rate ratios of tick-borne encephalitis per year, from 2015 to 2023, in Denmark, with 2015 as the reference, accounting for age and sex distribution in the population


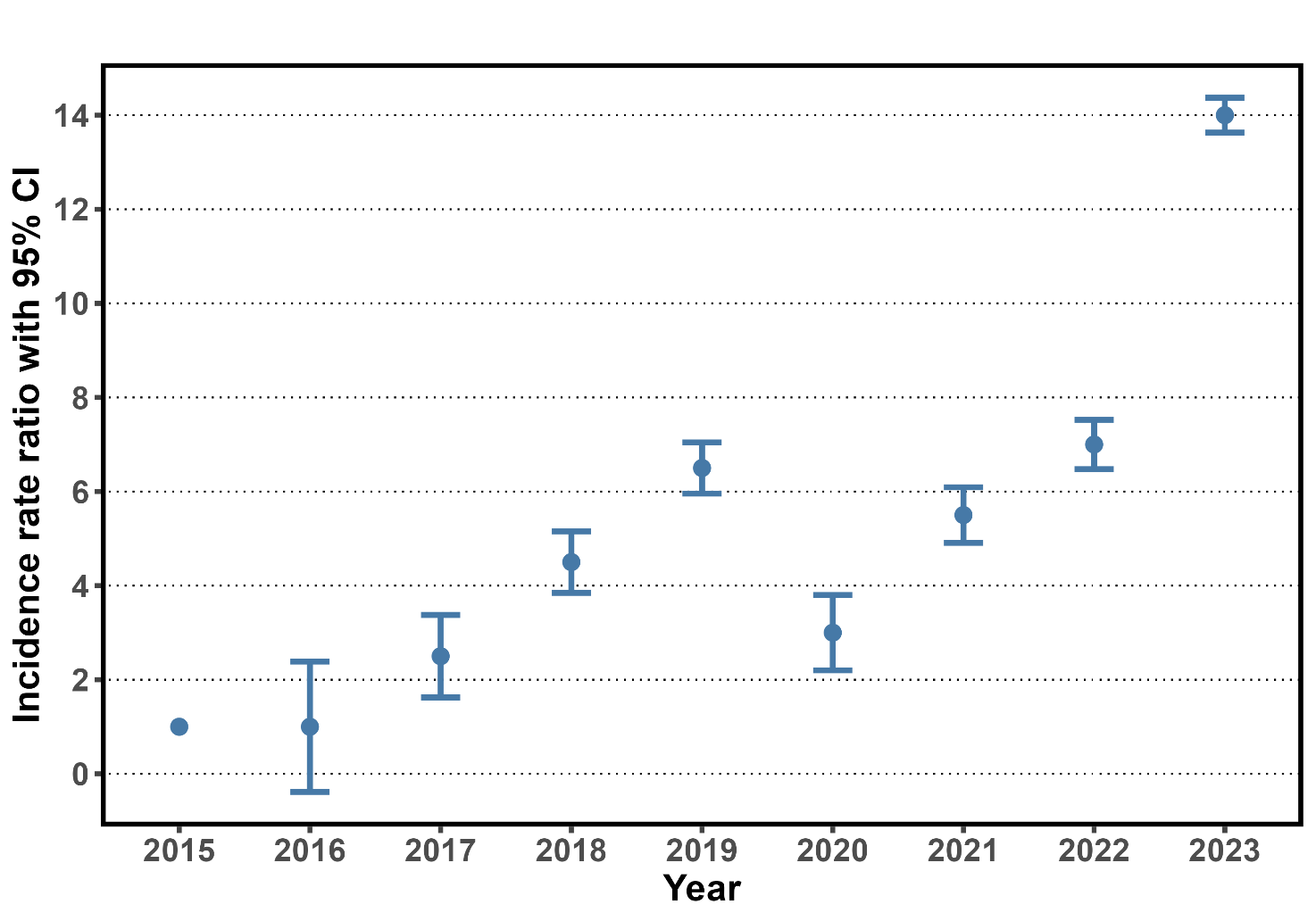


**Supplementary Figure 2.** Age percentage of all persons tested for tick-borne encephalitis, from 2015 to 2023, in Denmark. Every tested person only appears once per calendar year


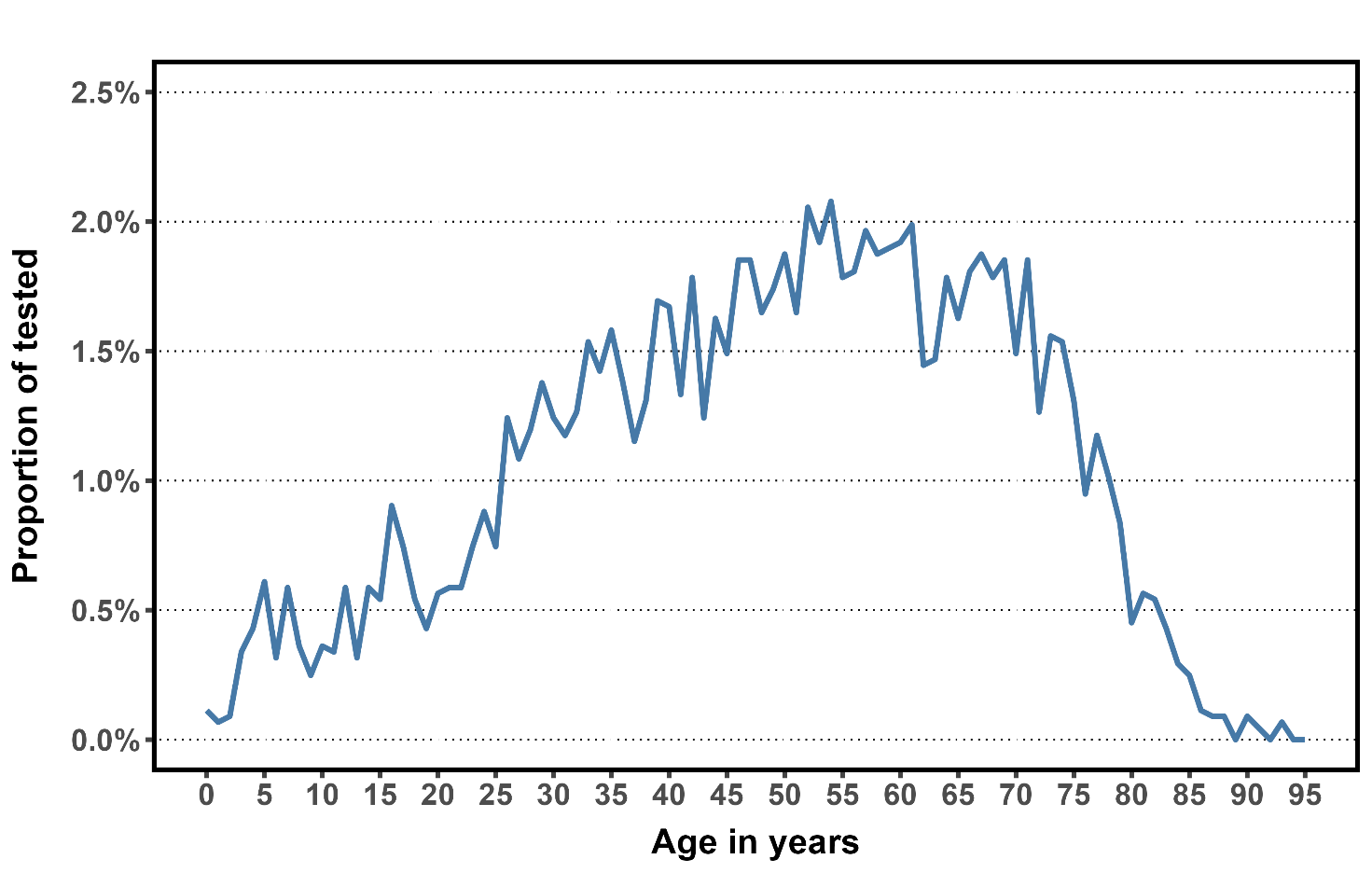

Supplement: Supplementary file 1 — Supplementary file1 (DOCX 193 KB) [file 415_2025_12986_MOESM1_ESM.docx]
